# Efficacy of Six-Week Extended-Dose Nevirapine Varies by Infant Birth Weight with Greatest Relative Efficacy in Low Birth Weight Infants

**DOI:** 10.1371/journal.pone.0162979

**Published:** 2016-09-30

**Authors:** Nikhil Gupte, Aarti Kinikar, Katherine N. McIntire, Ramesh Bhosale, Sandesh Patil, Nishi Suryavanshi, Vidya Mave, Vandana Kulkarni, Robert C. Bollinger, Amita Gupta

**Affiliations:** 1 Department of Medicine, Johns Hopkins University School of Medicine, Baltimore, Maryland, United States of America; 2 Byramji Jeejeebhoy Government Medical College-Johns Hopkins University HIV Clinical Trials Unit, Biramji Jeejeebhoy Government Medical College, Pune, Maharashtra, India; 3 Byramji Jeejeebhoy Government Medical College and Sassoon General Hospitals, Pune, Maharashtra, India; 4 Johns Hopkins Bloomberg School of Public Health, Baltimore, Maryland, United States of America; Centers for Disease Control and Prevention, UNITED STATES

## Abstract

Latest World Health Organization guidelines recommend weight-based nevirapine prophylaxis for all HIV-exposed infants in resource-limited settings, yet low birth weight (LBW) infants (< 2500 g) have been understudied. Using data from the NIH-funded India six-week extended-dose nevirapine (SWEN) study, a randomized clinical trial of SWEN versus single-dose nevirapine (SD) for prevention of breast-milk HIV-1 transmission, we examined the relative impact of SWEN among 737 mother-infant pairs stratified by infant birth weight. Birth weight groups were defined as very LBW (VLBW) ≤ 2000 g, moderate LBW (MLBW) >2000 g and ≤ 2500 g, and normal birth weight (NBW) > 2500 g. Outcomes were HIV-1 infection, HIV-1 infection or death by 12 months, and severe adverse events (SAEs). The Kaplan-Meier method was used to estimate probability of efficacy outcomes in birth weight groups, and differential effects of SWEN by birth weight group were examined using Cox proportional hazards models adjusting for independent risk factors for HIV maternal-to-child transmission and significant covariates. Among 50 VLBW, 249 MLBW, and 433 NBW infants, 50% were randomized to SWEN; median gestational age was 36, 38 and 38 weeks, respectively; and there was no difference in breastfeeding duration (p = 0.99). Compared to SD: SWEN-treated VLBW had lower estimates of HIV-1 infection (13% vs. 38%, p = 0.004) and HIV-1 infection or death (13% vs. 41%, p = 0.002); SWEN-treated MLBW had lower estimated HIV-1 infection (13% vs. 17%, p = 0.042); and efficacy endpoints were similar by treatment arm in NBW. In multivariate analysis, SWEN was associated with reduced risk of HIV-1 infection or death by 83% (p = 0.03) in VLBW versus 45% (p = 0.05) in MLBW. SAE frequency was similar by treatment arm in VLBW (68% vs. 76%, p = 0.53) and MLBW (37% vs. 36%, p = 0.93). SWEN may safely increase HIV-free survival among HIV-exposed LBW infants with greatest protective advantage among infants ≤ 2000 g.

## Introduction

Low birth weight (LBW), defined by the World Health Organization (WHO) as birth weight less than 2500 g, is a significant public health issue in resource-limited settings, particularly in Sub Saharan Africa and South Asia where the estimated annual incidence is 14% and 28–31%, respectively, and the HIV burden among women of reproductive age is high [1,2]. Maternal HIV infection is an independent risk factor for LBW [3,4], and LBW infants of HIV-infected women are at increased risk for mother-to-child transmission (MTCT) of HIV and death [5,6,7]. Amidst a rapidly evolving landscape, optimizing maternal and infant drug regimens for prevention of MTCT (PMTCT) of HIV infection remains a research priority. LBW infants, however, have been understudied.

Most recent WHO HIV PMTCT guidelines recommend lifelong combination antiretroviral treatment (ART) for pregnant and breastfeeding HIV-infected women (national PMTCT programme Option B or B+) and six weeks of daily weight-based nevirapine for all breastfed, HIV-exposed infants [8,9]. To date, several trials support extended nevirapine prophylaxis in infancy to prevent breast-milk MTCT of HIV infection [6,10–15]. However, safety and efficacy data in LBW infants are limited as most studies have either excluded or enrolled LBW infants in small numbers [6,11,15–20]. Notably, based on their relative physiologic immaturity, LBW infants may metabolize and respond differently to nevirapine than normal birth weight infants. Specific cytochrome P-450 enzymes isoforms with varying activity levels are differentially expressed throughout development, which leads to differences in nevirapine elimination in fetuses, neonates, and throughout infancy [21,22]. Thus, the impact of extended nevirapine prophylaxis may vary by infant birth weight and optimal dosing may not be known.

The present study aims to assess whether the impact of six-week extended-dose nevirapine (SWEN) prophylaxis varies with infant birth weight. The India SWEN study, a randomized clinical trial of SWEN versus single-dose nevirapine (SD) for PMTCT of HIV-1 infection via breast milk, presents a unique opportunity as LBW infants comprise approximately 40% of the study population, infants were randomized to treatment arm at birth, and HIV-1 transmission was assessed through age 12 months [10,12]. We report our secondary safety and efficacy analyses of the India SWEN study stratified by infant birth weight.

## Materials and Methods

### Study population and India SWEN study design

The secondary analysis study population included breastfed infants of HIV-infected pregnant women who participated in the India SWEN study (ClinicalTrials.gov number NCT00061321), a National Institutes of Health (NIH)-funded phase III randomized controlled trial comparing SWEN and SD to prevent breast-milk MTCT of HIV-1 infection. The participants enrolled in the India SWEN trail provided a written informed consent for trial participation. The primary outcome was HIV-1 transmission at 6 months; secondary endpoints included the composite outcome of HIV-1 transmission or death by 12 months as well as infant mortality at 12 months.

The SWEN trial methods and procedures have been previously described [10]. In brief, the study site was a large, urban public teaching hospital (Sassoon Hospital affiliated with Byramji Jeejeebhoy Government Medical College [BJGMC]) serving a semi-urban and rural population in Pune, Maharashtra, India. Between August 2002 and September 2007, HIV-infected women who intended to breastfeed were enrolled from the third trimester to within 24 hours of delivery. All infants received SD (2 mg/kg) at birth and were then randomized to receive SWEN plus multivitamins or multivitamins alone. Infants assigned to the SWEN arm received 5 mg of oral nevirapine daily from age 8 days to 42 days. Mother-infant pairs were followed for 12 months postpartum through scheduled study visits on weeks 1, 2, 3, 4, 5, 6, 10, 14 and at months 6, 9, and 12; each visit included an infant feeding assessment, a clinical examination, and select laboratory investigations.

Infants were tested for HIV-1 infection within 48 hours of birth and at all scheduled study visits (except weeks 3 and 5) using a DNA polymerase chain reaction (PCR) assay. Positive PCR results were confirmed with a quantitative HIV-1 viral load assay. HIV-1 infection was defined as HIV-1 viral load > 5000 copies/mL, and infant HIV-1 infection status was externally quality-assured as previously described [10]. The India SWEN study methods were approved by: the Johns Hopkins University Institutional Review Board, the Pune Institutional Review board, and the BJGMC Ethics Committee.

### Birth Weight Group Definitions

For secondary analysis, infants were assigned to one of three birth weight groups, defined using the WHO birth weight categories for weight-based infant nevirapine prophylaxis dosing recommendations [9]: ≤ 2000 g defined as very LBW (VLBW); > 2000 g and ≤ 2500 g defined as moderate LBW (MLBW); and > 2500 g defined as normal birth weight (NBW). Infant birth weight was obtained immediately following delivery and was measured to the nearest 100 g using a standard weighing scale [10]. Prior to treatment randomization, study clinicians evaluated the fitness of each LBW infant.

### Secondary Analysis Endpoints

The primary safety endpoint was severe adverse events (SAE) defined as any Grade 3 or Grade 4 adverse event (clinical or laboratory) or Grade 2 or higher neutropenia. SAEs were documented and classified using the NIH Division of AIDS Toxicity Tables for Grading Severity of Adverse Experiences (April 1994 version). Primary efficacy endpoints were HIV-1 infection and HIV-1 infection or death at 12 months. Secondary endpoints included HIV-1 infection at six weeks and six months and all-cause infant mortality at 12 months.

### Statistical Methods

We performed an intention-to-treat (ITT) analysis stratified by birth weight group using STATA software (version 11.1). Baseline maternal and infant characteristics were summarized within each birth weight group using median and interquartile range (IQR) or frequency and compared across birth weight groups using a Kruskal-Wallis non-parametric [23], χ^2^, or Fisher’s exact test [24] as appropriate. Paired birth weight group comparisons were performed for covariates with a p-value < 0.05.

We assessed the differential safety of SWEN using Poisson regression analysis; risk ratios (RR) for SAEs were modeled within each birth weight group by including an interaction term for SWEN and birth weight group. We assessed the differential efficacy of SWEN using Cox proportional hazards models; hazards ratios (HR) for efficacy endpoints were modeled within each birth weight group by including an interaction term for SWEN and infant birth weight group in the model and using the infant birth weight group for the analysis as referent. Multivariate models were adjusted for known risk factors for HIV-1 MTCT and covariates with p-values < 0.1 in univariate analysis. P-values ≤ 0.05 were deemed statistically significant.

A Kaplan-Meier non-parametric product limit estimator was used to estimate probability and 95% confidence interval (CI) of HIV-1 transmission, HIV-1 transmission or death by 12 months, and infant mortality by 12 months. Infants were censored at the earliest among study termination, last HIV-1 infection determination or death. The analysis was performed separately for SWEN and SD and stratified by infant birth weight group. Efficacy endpoints were compared by treatment arm using a log-rank test.

The ITT analysis was repeated as a modified ITT analysis, excluding infants with a positive test for HIV-1 infection within 48 hours of birth. Because LBW often occurs among premature infants (defined as gestational age <37 weeks), the differential impact of SWEN was also examined by gestational age (defined by last menstrual period and sonography).

## Results

### Study Population Characteristics

Among 737 mother-infant pairs enrolled in the India SWEN study, five infants were excluded due to missing birth weight data. The analysis included 732 infants with a median birth weight (IQR) of 2600 g (2400–3000). Birth weight groups were similar among breastfeeding duration, proportion of infants randomized to SWEN, median maternal age, maternal use of highly active antiretroviral therapy, and median maternal CD_4_ count at delivery (Table 1). VLBW had lower maternal antepartum zidovudine use compared to MLBW (8% vs. 31%, p = 0.001) and the lowest proportion of mothers who received intrapartum nevirapine relative to MLBW and NBW (30% vs. 61% vs. 73%, p<0.001). LBW groups (VLBW and MLBW) were similar among median infant gestational age (p = 0.09); male sex (p = 0.88); median maternal HIV-1 viral load at delivery (p = 0.86); maternal education less than primary level (p = 0.94); and maternal occupation of housewife (p = 0.08). Compared to LBW groups, NBW had a greater median infant gestational age, and NBW mothers had a lower median HIV-1 viral load at delivery (4.11 vs. 3.98 vs. 3.56 log_10_copies/mL, p<0.001), a higher level of education, and were more likely to be housewives.

**Table 1:** Baseline Characteristics by Infant Birth Weight Group.

	Overall	VLBW	MLBW	NBW	*P*
Mother-infant pairs, n (%)	732 (100)	50 (7)	249 (34)	433 (59)	
Maternal characteristics					
Age, median (IQR), y	23 (21–25)	24 (21–26)	22 (21–25)	23 (21–25)	0.20
Less than primary education,^a^ n (%)	292 (40)	23 (46)	113 (45)	156 (36)	0.04
Housewife,^a^^,^^b^ n (%)	591 (81)	33 (66)	193 (78)	365 (84)	0.002
Primigravida, n (%)	247 (34)	15 (30)	89 (36)	143 (33)	0.67
Gestational age,^a^^,^^b^^,^^c^ median (IQR), weeks	39 (38–40)	36 (34–37)	38 (37–39)	39 (38–40)	<0.001
Normal vaginal delivery, n (%)	587 (80)	41 (82)	198 (80)	348 (80)	0.94
Antepartum zidovudine,^b^^,^^c^ n (%)	247 (34)	4 (8)	78 (31)	165 (38)	<0.001
Intrapartum nevirapine,^a^^,^^b^^,^^c^ n (%)	484 (66)	15 (30)	153 (61)	316 (73)	<0.001
HAART,^a^ n (%)	61 (8)	6 (12)	27 (11)	28 (6)	0.07
CD_4_ count, median (IQR), cells/mm^3^	466 (314–650)	504 (252–632)	445(301–613)	472 (328–667)	0.24
HIV-1 viral load,^a^^,^^b^ median (IQR), log_10_copies/mL	3.7 (2.9–4.5)	4.11 (3.30–4.76)	3.98 (3.14–4.70)	3.56 (2.72–4.38)	<0.001
Infant characteristics					
Male sex,^a^ n (%)	388 (53)	24 (48)	116 (47)	248 (57)	0.02
Birth weight,^a^^,^^b^^,^^c^ median (IQR), kg	2.6 (2.4–3.0)	1.98 (1.75–2.00)	2.40 (2.25–2.50)	2.90 (2.70–3.10)	<0.001
Gestational age,^a^^,^^b^ median (IQR), weeks	38 (38–38)	36 (35–37)	38 (37–38)	38 (38–38)	<0.001
SWEN, n (%)	364 (50)	25 (50)	125 (50)	214 (49)	0.98
Breastfeeding duration, n (%)					
< 4 months	403 (55)	27 (54)	138(55)	238 (55)	
4–6 months	71 (10)	4 (8)	23(9)	44 (10)	0.99
≥ 6 months	258 (35)	19 (38)	88(35)	151 (35)	

Abbreviations: HAART, highly active antiretroviral therapy; IQR, interquartile range; MLBW, moderate low birth weight (>2000 g and ≤ 2500 g); NBW, normal birth weight (> 2500 g); SWEN, six-week extended-dose nevirapine; VLBW, very low birth weight (≤ 2000 g).

^a^ P < 0.05 for comparison between MLBW and NBW.

^b^ P < 0.05 for comparison between VLBW and NBW.

^c^ P < 0.05 for comparison between VLBW and MLBW.

### Safety

A total of 409 SAEs were documented among 259 (35%) infants. Table 2 shows the proportion of infants with at least one SAE (SAE frequency), the total number of SAEs, and SAE classification for each treatment arm stratified by infant birth weight group. Overall, Anemia and All-cause hospitalization were most common, and total number of SAEs was similar by treatment arm across birth weight groups. Overall SAE frequency was highest within VLBW compared to MLBW (72% vs. 37%, p<0.001) and NBW (72% vs. 30%, p<0.001), but similar between MLBW and NBW (p = 0.06). By treatment arm, SAE frequency was similar in SWEN and SD within VLBW (68% vs. 76%, p = 0.53), MLBW (37% vs. 36%, p = 0.93), and overall (33% vs. 38%, p = 0.13), but lower in SWEN within NBW (26% vs. 35%, p = 0.054). In multivariate analysis adjusted for significant covariates, SWEN was associated with lower relative risk of SAEs within each infant birth weight group, yet RRs were not statistically significant (Table 3).

**Table 2:** Safety Endpoints by Treatment Arm and Infant Birth Weight Group.

	Overall (n = 732)			VLBW (n = 50)			MLBW (n = 249)			NBW (n = 433)		
	SD	SWEN	*P*	SD	SWEN	*P*	SD	SWEN	*P*	SD	SWEN	*P*
Safety Endpoint^a^												
Infants with any SAE, n (%)	140 (38)	119 (33)	0.13	19 (76)	17 (68)	0.53	45 (36)	46 (37)	0.93	76 (35)	56 (26)	0.054
Number of SAEs												
Total	223	186		44	32		74	58		105	96	
All Grade 3^b^	119(53)	115(62)	0.01	29(66)	25(78)	0.20	31(42)	34(59)	0.053	59(56)	56(58)	0.77
All Grade 4^b^	94(42)	62(33)	0.06	13(30)	4(13)	0.08	41(55)	20(34)	0.02	40(38)	38(40)	0.77
Grade 2 or higher neutropenia	10(4)	9(5)	0.63	2(5)	3(9)	0.49	2(3)	4(7)	0.28	6(6)	2(2)	0.10
SAE Type^c^												
Rash	0	0		0	0		0	0		0	0	
Anemia	31	27		11	7		7	7		13	13	
Neutropenia ^c^	10	9		2	3		2	4		6	2	
Elevated LFTs	13	6		3	3		3	1		7	2	
Other abnormal lab values	7	5		2	0		0	3		5	2	
Gastrointestinal conditions	7	7		0	1		1	3		6	3	
Respiratory conditions	3	7		0	0		1	4		2	3	
Other conditions	14	11		3	2		3	2		8	7	
All-cause hospitalization	119	105		18	16		49	29		52	60	
Neonatal or infant death	19	9		5	0		8	5		6	4	

Abbreviations: LFT, liver function test; MLBW, moderate low birth weight (>2000 g and ≤ 2500 g); NBW, normal birth weight (> 2500 g); SAE, severe adverse event; SD, single-dose nevirapine; SWEN, six-week extended-dose nevirapine; VLBW, very low birth weight (≤ 2000 g).

^a^ Severe adverse event defined as any grade 3 or 4 adverse event or grade 2 or higher neutropenia using the National Institutes of Health Division of AIDS Toxicity Tables for Grading Severity of Adverse Experiences, version April 1994.

^b^ Neutropenia excluded.

^c^ Grade 3 and Grade 4 combined, except for Neutropenia, which includes Grade 2 and higher.

**Table 3:** Estimated Risk and 95% Confidence Intervals of Study Endpoints for SWEN relative to SD by Infant Birth Weight Group Using Poisson Regression and Cox Proportional Hazards Analyses.

	VLBW		MLBW		NBW	
	Unadjusted	Adjusted	Unadjusted	Adjusted	Unadjusted	Adjusted
Study Endpoint						
Incidence of SAE^a^	0.67 (0.38, 1.19) [0.17]	0.65 (0.36, 1.16) [0.15]	0.82 (0.63, 1.06) [0.13]	0.82 (0.63, 1.07) [0.14]	0.88 (0.70, 1.11) [0.29]	0.87 (0.69, 1.10) [0.24]
HIV-1 transmission^b^						
6 weeks	0.03 (0.003, 0.33) [0.004]	0.05 (0.004, 0.71) [0.03]	0.40 (0.16, 0.95) [0.04]	0.48 (0.20, 1.18) [0.11]	0.88 (0.40, 1.97) [0.76]	1.01 (0.44, 2.30) [0.98]
6 months	0.12 (0.02, 0.67) [0.02]	0.17 (0.03, 1.09) [0.06]	0.62 (0.31, 1.24) [0.18]	0.67 (0.33, 1.37) [0.28]	0.87 (0.46, 1.66) [0.68]	0.97 (0.50, 1.88) [0.94]
12 months	0.15 (0.03, 0.70) [0.02]	0.19 (0.03, 1.04) [0.06]	0.59 (0.31, 1.13) [0.11]	0.62 (0.32, 1.20) [0.16]	0.93 (0.53, 1.62) [0.79]	1.03 (0.59, 1.83) [0.90]
HIV-1 transmission or death at 12 months^b^	0.15 (0.03, 0.67) [0.01]	0.17 (0.03, 0.85) [0.03]	0.57 (0.32, 1.01) [0.06]	0.55 (0.30, 1.00) [0.05]	0.79 (0.46, 1.36) [0.40]	0.89 (0.51, 1.54) [0.68]
Mortality at 12 months^b^	NA^c^	NA^c^	0.45 (0.15, 1.34) [0.15]	0.45 (0.14, 1.38) [0.16]	0.55 (0.15, 2.0) [0.36]	0.60 (0.16, 2.23) [0.44]

Abbreviations: HIV-1, human immunodeficiency virus type 1; MLBW, moderate low birth weight (>2000 g and ≤ 2500 g); NA, not analyzed; NBW, normal birth weight (> 2500 g); SAE, severe adverse event; SD, single-dose nevirapine; SWEN, six-week extended-dose nevirapine; VLBW, very low birth weight (≤ 2000 g).

^a^ Data presented as Relative Risk (95% confidence interval) [P-value]. Analysis adjusted for maternal age, maternal education, maternal CD4 count at delivery, maternal HIV-1 viral load at delivery, intrapartum nevirapine, infant gender, infant gestational age, and breastfeeding duration.

^b^ Data presented as Hazard Ratio (95% confidence interval) [P-value]. Analysis adjusted for maternal age, maternal education, maternal CD4 count at delivery, maternal HIV-1 viral load at delivery, antenatal zidovudine, intrapartum nevirapine, infant gestational age, and breastfeeding duration.

^c^ SWEN had zero deaths in VLBW

### Efficacy

**Fig 1:**
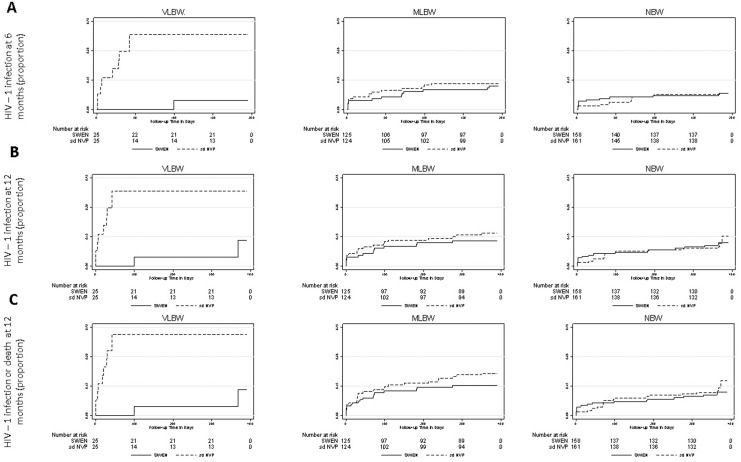
Kaplan-Meier plots of risk of HIV-1 infection (A and B) and HIV-1 infection or death (C) by treatment arm stratified by infant birth weight group. (A) HIV-1 infection by 6 months. (B) HIV-1 infection by 12 months. (C) HIV-1 infection or death by 12 months. Estimated risk of each efficacy outcome is shown for infants randomized to six-week extended-dose nevirapine (SWEN) and single-dose nevirapine (SD) as a solid line and dashed line, respectively, within each infant birth weight group. Birth weight groups were defined as: very low birth weight (VLBW ≤ 2000g); moderate low birth weight (MLBW >2000 g and ≤ 2500 g); and normal birth weight (NBW > 2500 g). Infants were tested for HIV-1 infection at birth, at weeks 1, 2, 4, 10 and 14, and at months 6, 9 and 12. Superior efficacy of SWEN relative to SD is indicated exclusively in VLBW and MLBW with greatest relative SWEN efficacy in VLBW.

Kaplan-Meier estimates of the risk of HIV-1 infection by 6 and 12 months and HIV-1 infection or death by 12 months were compared by treatment arm within each birth weight group (Fig 1). In VLBW, SWEN was associated with lower risk of HIV-1 infection by 6 months (5% vs. 38%, p = 0.004) and 12 months (13% vs. 38%, p = 0.004), lower risk of HIV-1 infection or death by 12 months (13% vs. 41%, p = 0.002), and lower risk of all-cause infant mortality at 12 months (0% vs. 38%, p = 003). Except for lower risk of HIV-1 infection by 12 months in SWEN-treated MLBW (13% vs. 17%, p = 0.04), estimated risk of efficacy endpoints was similar by treatment arm within MLBW and NBW, including mortality (MLBW p = 0.47; NBW p = 0.76) (data not shown but available upon request),

Results from Cox proportional hazards analysis for differential efficacy of SWEN by infant birth weight are shown in Table 3. Analysis adjusted for maternal age, maternal education, antepartum zidovudine, intrapartum nevirapine, maternal CD_4_ count, maternal HIV-1 viral load, breastfeeding duration, and infant gestational age. Within VLBW: relative to SD, SWEN was associated with reduced risk of HIV-1 transmission at 6 weeks (adjusted Hazards ratio [aHR] 0.05; 95% confidence interval [CI] 0.004, 0.71; p = 0.03); decreased individual infant risk of HIV-1 transmission at 6 months and 12 months by 83% (p = 0.06) and 81% (p = 0.06), respectively; and reduced risk of HIV-1 transmission or death at 12 months (aHR 0.17; 95% CI 0.03, 0.85; p = 0.03). In MLBW, SWEN was associated with reduced risk of HIV-1 transmission or death at 12 months (aHR 0.55; 95% CI 0.3, 1.0; p = 0.05), yet was not associated with significantly reduced hazards of any efficacy endpoint within NBW or a mortality benefit in MLBW or NBW (Table 3).

The results of the modified ITT analysis are not shown but were consistent with the ITT analysis (data available upon request). Additional analyses did not indicate a differential impact of SWEN by gestational age (data not shown but available upon request).

## Discussion

Impact of extended nevirapine prophylaxis has been largely understudied in LBW infants. This secondary analysis of the India SWEN study stratified by infant birth weight documents the relative safety and superior efficacy of SWEN versus SD in HIV-exposed LBW infants and that SWEN efficacy varies with infant birth weight. While SWEN was found to be as safe as SD in LBW study groups and protective to reduce HIV-1 transmission across birth weight groups, our analysis indicates a protective advantage of SWEN exclusively among LBW infants with greatest impact in the lowest LBW infants. Compared to SD, SWEN was associated with significantly lower risk of HIV-1 infection by 12 months exclusively in MLBW (13% vs. 17%, p = 0.04) and VLBW (13% vs. 41%, p = 0.002), yet only SWEN-treated VLBW infants had significantly lower risk of all efficacy endpoints, including mortality at 12 months (0% vs. 38%, p = 0.03), and greater differential efficacy of SWEN compared to other birth weight groups (Fig 1), including 83% (p = 0.03) reduction in risk of HIV-1 transmission or death in VLBW vs. 45% (p = 0.05) in MLBW. Overall our results support WHO guidelines, which recommend higher weight-based infant nevirapine dosing than SWEN in infants > 2000 g [9], yet include new data that contribute to understanding the impact of extended nevirapine prophylaxis in LBW infants.

Relative safety and/or superior efficacy of various extended nevirapine prophylaxis regimens have been reported in predominantly normal birth weight infants, including the separate primary analyses of the Post-Exposure Prophylaxis of the Infant (PEPI), SWEN, Breastfeeding Antiretrovirals and Nutrition (BAN) and HIV Prevention Trials Network (HPTN) 046 trials [6,10,11, 13–15]. Our analysis, however, is among the first to assess the safety and efficacy of an extended nevirapine regimen in LBW infants. Although the lowest LBW group had the largest proportion of infants with at least one SAE, SAE frequency was similar in the SWEN and SD treatment arms within both LBW study groups (VLBW and MLBW). In addition, total number of SAEs was similar by treatment arm independent of birth weight group, and multivariate analysis did not associate SWEN with significantly lower relative risk of SAEs within any birth weight group after adjusting for significant covariates. Given the small sample size of the VLBW study group, we would expect SAEs to be rare. Despite this limitation, our analysis suggests that SWEN is as safe as SD among LBW infants.

Regarding efficacy, our analysis indicates a protective advantage of SWEN prophylaxis over SD among LBW infants with greatest relative SWEN efficacy in the lowest LBW group. SWEN was associated with significantly lower estimated risk of all HIV-1 transmission endpoints than SD exclusively in VLBW with greater relative efficacy compared to other birth weight groups (Fig 1). Notably, the protective effect of SWEN in VLBW remained in multivariate analysis after adjusting for independent risk factors for HIV MTCT, including maternal viral load, breastfeeding duration, and maternal HAART, as well as significant covariates, such as antenatal zidovudine, making it highly likely to be attributable to SWEN. In contrast, despite evidence for a protective advantage in the higher LBW group (MLBW), namely significantly lower risk of HIV-1 infection and reduced hazards of HIV-1 transmission or death, our analysis indicates that the overall effect of SWEN on HIV-1 transmission is similar to SD in MLBW (Fig 1). Moreover, our analysis provides no evidence to suggest a mortality benefit in infants with birth weight > 2000 g or a significant protective advantage of SWEN in infants > 2500 g. Acknowledging that stratified analysis limits comparisons across strata and that the analysis is underpowered in NBW, our results suggest greatest impact of SWEN in infants with birth weight ≤ 2000 g and lesser to no protective advantage in higher birth weight infants.

Few studies have evaluated extended nevirapine prophylaxis in LBW infants or by infant birth weight. In comparison to separate primary analysis of the India SWEN study, which associates SWEN with a 39% reduction in risk of HIV-1 transmission or death at 12 months (p = 0.020)[12], our analysis suggests that the lowest LBW infants may drive the superior efficacy of SWEN reported in the overall study population. That SWEN efficacy varies with infant birth weight may be explained by developmental differences in nevirapine elimination [21]. While our birth weight analysis does not include pharmacokinetic data, our finding of greatest SWEN impact in the lowest LBW group is consistent with the one study to date that has reported pharmacokinetic data for infant nevirapine prophylaxis in LBW infants. This South African study found similar plasma nevirapine concentrations among term and preterm infants following single-dose nevirapine (2 mg/kg) at birth; however, among preterm infants, small for gestational age infants had a larger area under the plasma concentration time curve (p = 0.006) and lower clearance (p<0.0001) than appropriate for gestational age infants [22]. It is feasible that relatively reduced nevirapine elimination may correspond to greater relative SWEN efficacy in VLBW compared to higher birth weight infants, which may have implications for nevirapine dosing.

Our results suggest that SWEN dosing may be sufficient to reduce HIV-1 transmission in infants with birth weight < 2000 g, yet may be sub-optimal in higher birth weight infants. Overall, this implication is consistent with latest WHO HIV PMTCT guidelines. Supported by data from the PEPI, BAN and HPTN 046 trials, WHO guidelines currently recommend weight-based six-week extended nevirapine dosing that is two to three times higher than SWEN among infants with birth weight > 2000 g [6,9,11,13–15]. However, among infants with birth weight < 2000 g, a considerably less well studied population, the WHO suggested starting dose (2 mg/kg) is lower than SWEN (5 mg) [9]. Interestingly, a pooled analysis of the SWEN, BAN and HPTN trials, which have assessed impact of extended nevirapine courses lasting six weeks, 28 weeks and six months, respectively, correlates longer nevirapine courses with greater efficacy [25]. Although current weight-based infant nevirapine prophylaxis regimens may better account for pharmacokinetic and pharmacodynamic differences at birth and throughout infancy, without pharmacokinetic studies and clinical trials that assess potential differential impacts of regimens by infant birth weight and specifically in LBW infants, optimal regimens may still not be known.

Further, the overall benefit of SWEN among breastfed HIV-exposed infants needs to be determined in light of 2015 WHO guidelines, which recommend Option B+ (lifelong ART for HIV-infected pregnant and breastfeeding women) for the prevention of HIV MTCT [26]. Recent studies from Malawi and Ghana have reported substantial decreases in vertical HIV transmission to breastfed infants with Option B+ [27,28], and a study from Nigeria reported virtually no HIV MTCT among breastfed infants who also received SWEN prophylaxis [29]. Elimination of HIV MTCT may be achievable with Option B+; however, despite being one of the WHO’s 22 global targets for scale-up of PMTCT interventions, India has very recently implemented Option B+ on a national level. Notably, studies from India and Ethiopia have reported increased risk of drug-resistant virus among infants receiving SWEN [30,31]. The benefit of infant SWEN prophylaxis remains relevant, albeit not without risk, and warrants further study to define its role in the era of Option B+. There remain HIV-infected women who are not virally suppressed during pregnancy and/or labor and these women are often the ones who have high rates of LBW infants and thus most likely to benefit from SWEN.

## Conclusions

Our secondary analysis of the India SWEN study stratified by infant birth weight documents that SWEN efficacy varies with infant birth weight and provides evidence that SWEN prophylaxis may safely increase HIV-free survival among HIV-exposed LBW infants with greatest protective impact among infants with birth weight ≤ 2000 g. Our results support higher birth weight-based nevirapine dosing in infants with birth weight > 2000 g as currently recommended by WHO guidelines, yet highlight that optimal dosing may still not be known. To optimize dosing and to provide safe and effective drug therapy during infancy, there is a clear need for clinical trials that are prospectively designed to include LBW infants and that examine differential effects of pharmacologic interventions on clinical endpoints by infant birth weight.
